# Protein Adequacy Is Primarily a Matter of Protein Quantity, Not Quality: Modeling an Increase in Plant:Animal Protein Ratio in French Adults

**DOI:** 10.3390/nu9121333

**Published:** 2017-12-08

**Authors:** Erwan de Gavelle, Jean-François Huneau, Clélia M. Bianchi, Eric O. Verger, François Mariotti

**Affiliations:** 1 UMR PNCA, AgroParisTech, INRA, Université Paris-Saclay, 75005 Paris, France; erwan.degavelle@agroparistech.fr (E.d.G.); jean_francois.huneau@agroparistech.fr (J.-F.H.); clelia.bianchi@agroparistech.fr (C.M.B.); 2 NUTRIPASS, IRD, Université Montpellier, SupAgro, 34394 Montpellier, France; eric.verger@ird.fr

**Keywords:** plant protein, animal protein, protein adequacy, amino acid adequacy, modeling study

## Abstract

A downward trend in animal protein (AP) intake has been observed in western countries over the last decade and the effects of such a transition on protein adequacy remain debatable. Using the probability approach and diet modeling with data on 1678 adults from a representative French national dietary survey, we studied the sensitivity of the adequacy of protein and amino acid intakes to changes in animal:plant protein. We simulated the gradual substitution of AP with different mixtures of plant protein (PP), containing various proportions of PP already consumed and legumes, nuts and seeds (LNS). We found that protein and amino acid intakes met dietary requirements in virtually the entire population studied. Up to 50% of PP in diets, protein and amino acid intakes were adequate in all models. From 50%, protein inadequacy was primary due to protein quantity, and from 70%, to protein quality (as lysine inadequacy). The introduction of LNS in the mixture substituting AP led to adequate protein intakes for higher percentages of PP. An increase in PP based on the current pattern of plant protein sources, low in protein:energy, could lead to inadequate protein intake, but the contribution of LNS ensures the safety of a further transition.

## 1. Introduction

Dietary protein is indispensable to deliver the nitrogen and amino acids required to meet metabolic demands, and particularly the renewal of body proteins. The results of nitrogen balance studies were used to define a protein requirement to be met using “good quality” dietary protein [1,2]. Nine amino acids (AA) which cannot be synthesized de novo in the body, or at a sufficient rate, are classified as indispensable (IAA). Dietary requirements for IAA have been set from the results of tracer-based studies. Protein and IAA requirements are used conjointly to define the quality of a given dietary protein [1]. A “good quality” protein is therefore one with high digestibility (as is the case for most animal proteins) that is rich in all the individual IAA required, i.e., a protein that will supply sufficient IAA to cover requirements when consumed at a level that complies with the global protein requirement. In this respect, the differences between animal and plant sources of protein have been highlighted: Plant protein sources tend to contain less overall protein, and cereals have a lower lysine content as a percentage of total protein [3] and slightly poorer digestibility [4] than animal protein [5]. This has given rise to concerns that a higher or a predominant intake of plant protein might lead to inadequate intake of protein and IAA when compared to requirements. 

In Europe and the USA, dietary protein mainly comes from animal sources (between 55% and 71%, depending on the country) and particularly from red meat (which contributes between 16% and 35% to animal protein intake). Cereals are the principal contributors to plant protein intake (between 40% and 70% of plant protein) [6,7,8]. However, in western countries, the intake of animal products, and particularly meat, has decreased since the early 2000s (for instance, the mean meat intake has decreased by ~10% from 1998 to 2015 in France) [9]. This trend can be explained by the economic crisis, changes to household structure [9] and concerns about human health, animal welfare and the environment [10]. Furthermore, national guidelines tend to advise an increase in plant protein intake (from legumes and nuts in particular) and limitations on some animal proteins (cured and processed meats, red meat) [11,12]. Current and future trends could therefore lead to an inversion of the plant:animal protein ratio.

The literature contains protein intake estimates in western countries and populations who consume various levels of plant protein [6,13,14,15,16]. However, only a few studies have assessed the adequacy of their usual protein intake (as compared to dietary protein requirements). These studies have shown no prevalence of an inadequate protein intake in France [14], almost no inadequacy among U.S. male adults and little inadequacy among U.S. females adults [13]. Using a modeling analysis, the U.S. Dietary Guidelines Advisory Committee reported that even people consuming high levels of plant-based protein would be able to meet their protein requirements [12]. Data on the dietary intake of IAA, and its adequacies, remain scarce. Amino acid intake was assessed in the USA in 1988–1994 [17] and intake and adequacies were assessed in Japan from 2002–2003 [18] and in France in 1999 (for lysine and methionine only) [19]. These assessments showed no prevalence of inadequate intake for any of the IAA. However, based on the current food intake, some studies have proposed that compliance with recommendations designed to reduce the consumption of animal-based products may cause problems in meeting the recommended intake of certain nutrients, including protein [7], and prospective modeling of a 100% increase in the dietary plant protein intake showed that this would lead to an inadequate protein intake in a significant proportion of the population [20]. 

The literature on protein and amino acid adequacy is limited and is based on studies performed several years ago. Based on the current trend towards modifying the plant:animal protein ratio, and the evolutions anticipated in the future, it is necessary to determine the extent to which intake of protein and amino acids (and notably lysine) are sufficient at present to meet requirements, to characterize their association with the plant:animal protein ratio and to study its sensitivity to probable ongoing and future changes in intake. 

## 2. Materials and Methods

### 2.1. Population and Dietary Data

The data used in this study were derived from the 7-day food records of adults involved in the second individual and national study on food consumption survey (INCA2) which was performed from 2006–2007 [21]. Adults over 65 years old were excluded because the reference intake in this population was estimated as being higher than the rest of the adult population by the French Food Agency (1.0 g/kg b.w./day instead of 0.8 g/kg b.w./day) [2]. Under and over-reporters were also excluded, based on a comparison between the reported energy intake and the basal metabolic rate, as estimated using Henry equations [22], and a cut off-value as defined by Black et al. [23], with physical activity levels of 1.4, 1.6 and 1.8 for little active (sedentary), moderately active and active lifestyles [24]. To ensure the representativeness of the sample, the data were weighted for unequal sampling probabilities and for differential non-responses by region, agglomeration size, age, sex, occupation of the household head, size of the household and season. The final sample contained 1678 adults (717 men and 961 females).

### 2.2. Energy, Protein and Amino Acid Compositions of the Diet

The nutrient contents of INCA2 foods were extracted from the 2016 CIQUAL (*Centre d’Information sur la Qualité des Aliments*—Centre for Information on Food Quality) database. Crude protein data (N × 6.25) were used for the protein content since protein requirements had been estimated from nitrogen balance studies [25].

As the amino acid contents of INCA2 foods were not available in the 2016 CIQUAL database, an amino acid database was compiled using published French analyses [26] and international databases [27,28,29,30]. The complete method is detailed in Method S1.

To take account of the poorer digestibility of plant protein, a 5% penalty was applied to the protein intake from plant protein food items before the protein and amino acid intakes and probability of inadequacy were calculated. This 5% difference was based on the set of real ileal protein digestibility studies in humans [4,31] by calculating the mean digestibility of the plant protein foods tested and that of animal protein foods, weighted by study sample sizes.

The percentages of animal and plant protein in each food item were obtained by assigning food items to both categories and breaking down the mixed food into ingredients from the recipes, as described in detail elsewhere [8].

### 2.3. Adequacy of Protein and Individual Amino Acid Intakes

Usual protein and amino acid intakes were determined using the Multiple Source Method [32,33]. The prevalence of the inadequacy of usual protein and amino acid intakes was estimated using the probability approach [34,35]. We took account of variability in the EAR (estimated average requirement) (CV = 12.5% for protein and amino acids) and used the CDF function of SAS 9.1 that computes the left cumulative distribution function of the normal distribution. The prevalence of inadequacy according to the probability approach produces the best estimate of the proportion of individuals in a population with intakes which do not meet their requirements [34,35]. 

The EAR values used in this study were those estimated by the FAO/WHO/UNU [36] (Table S1).

### 2.4. Simulations of Changes to the Plant:Animal Protein Ratio

**Figure 1:**
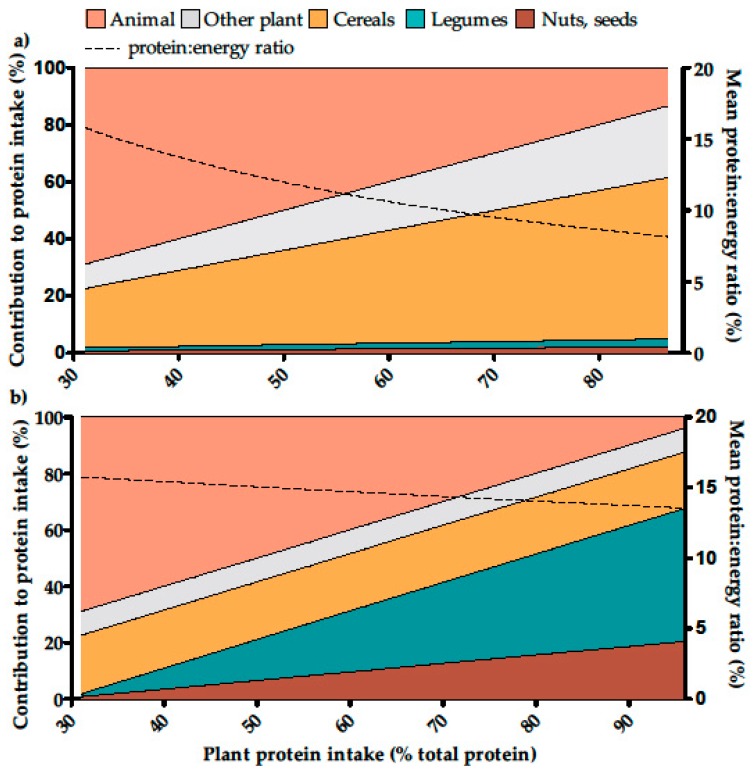
Contribution of animal protein and various plant sources (left axis) and protein:energy ratio (right axis) in the energy adjusted models of substitution of animal protein for plant foods in the French adult population (INCA2 study, *n* = 1678). (**a**) Animal protein was gradually substituted by the same amount of energy (without alcohol) from plant foods according to the current pattern of consumption in individuals (model A); (**b**) Animal protein was gradually substituted by the same amount of energy (without alcohol) from a mixture of legumes, nuts and seeds (model B). Intermediate models between A and B, i.e., models using 20%, 40%, 60% and 80% of a mixture of legumes, nuts and seeds in the combination used for the substitution are not shown. The highest plant protein level was not 100% due to the intake of mixed foods with both animal and plant protein sources.

We established four models for substitutions between animal and plant protein in the diet of each individual:

- Model P, a protein-adjusted model in which the animal protein was replaced with the same amount of plant protein using sources already consumed by each individual. The proportion of protein from each plant food within the total plant protein intake was kept constant, as was the proportion of protein from each animal food within the total animal protein intake. In this model, the total protein intake of each individual thus remained constant.
- Model A, an energy-adjusted model in which animal protein was replaced with the same amount of energy (without alcohol) intake from plant foods already consumed. The proportion of energy from each plant food within the total plant energy intake was kept constant, as was the proportion of protein from each animal food within the total animal protein intake. In this model, the total energy intake (without alcohol) of each individual thus remained constant (Figure 1a).
- Model B, an energy-adjusted model in which animal protein was replaced with the same amount of energy (without alcohol) from a mix of legumes, nuts and seeds. The mix was defined by the observed intake of legumes, nuts and seeds in the INCA2 study. For example, 1 g of beef steak protein (i.e., 3.6 g of beef steak) is replaced by 0.17 g of legumes protein (2.35 g of legumes) and 0.07g of nuts and seeds protein (0.34 g of nuts and seeds) (Figure 1b).
- Additionally, intermediate models combining model A and model B were designed. In these models, animal protein was replaced with the same amount of energy (without alcohol) from both plant foods already consumed and the mix of legumes, nuts and seeds in various proportions. The proportions of the mix of legumes, nuts and seeds in the substituting combination were 0% (i.e., same as Model A), 20% (model C_20_), 40% (model C_40_), 60% (model C_60_), 80% (model C_80_) and 100% (i.e., same as Model B).

Based on these models, we simulated a continuous increase in the mean plant protein percentage in the population and assessed the corresponding adequacy of resulting protein and IAA intake. 

Particular emphasis was placed on lysine in the results as this was found to be the limiting IAA (i.e., that with the highest prevalence of inadequacy) in the different models.

### 2.5. Statistical Analyses

Data are presented as means ± SDs. A linear regression model was used to determine the association between protein and amino acid intakes and the plant protein intake (as a percentage of total intake). Then, adjustments were made for age, sex, body mass index (BMI) and energy intake (without alcohol). Significance was set at *p* < 0.05. Statistical analyses were performed using SAS 9.1.3 (SAS Institute Inc., Cary, NC, USA).

## 3. Results

### 3.1. Protein and Amino Acid Intakes and Adequacy to Requirements

**Figure 2:**
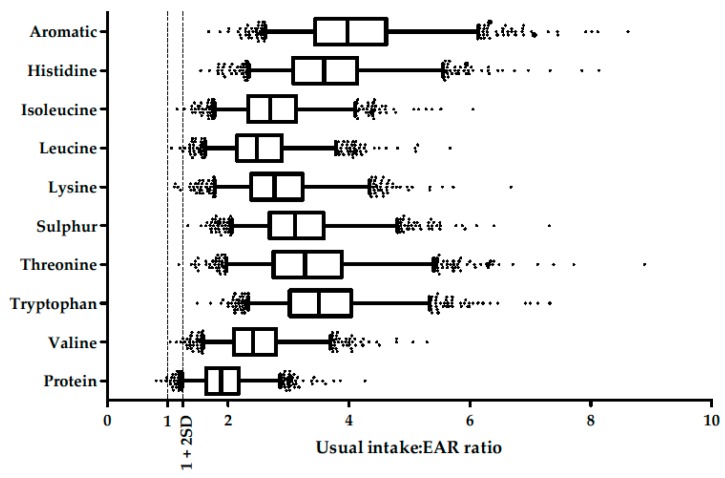
Usual intake:EAR ratio distribution of protein and amino acids in the French adult population (INCA2 study). Distribution of usual intakes of protein and IAA are represented using boxplots with median, 25th and 75th percentiles and whiskers drawn left to the 2.5th and right to the 97.5th percentiles. Points are individuals outside the 2.5th–97.5th percentile range. If the ratio between the usual intake and EAR of an individual was equal to 1, the probability of an adequate intake was 50% and if the ratio was 1 + 2SD, the probability of an adequate intake was 97.5%. The prevalence of inadequacy in the population was lower than 0.05% for all IAA and 0.31% for protein. EAR, Estimated average requirement; INCA2, Individual and National Consumption Survey 2 (*n* = 1678).

The mean protein intakes were 1.34 (±0.34) and 1.25 (±0.30) g/kg b.w./day (91 (±26) and 85 (±23) mg/kg b.w./day for lysine and 32 (±9) and 30 (±8) mg/kg b.w./day for methionine) in French adult men and women, respectively. The mean protein and amino acid intakes stratified by sex, age and BMI are presented in Tables S3 and S4. The prevalence of inadequacy of the usual intake was estimated as being less than 0.05% for all individual amino acids (Figure 2) and 0.31% for protein. 

### 3.2. Foods Contributing to Protein and Amino Acid Intakes

Animal and plant proteins contributed respectively 69% and 31% to the total protein intake, as previously reported [8] for both men and women. The main plant contributor of the total protein intake was cereals (21% in men, 20% in women), which accounted for 67% of the plant protein intake. The principal animal contributor of the total protein intake was meat (41% in men, 35% in women), which accounted for 59% of animal protein intake in men, and 51% in women. The lysine intake was mainly due to animal intake (84% in men, 82% in women), and particularly meat (51% in men, 43% in women). Cereals represented 9% of lysine intake in both men and women (Table 1).

### 3.3. Association between Protein and Lysine Adequacy and Dietary Plant Protein Intake

**Figure 3:**
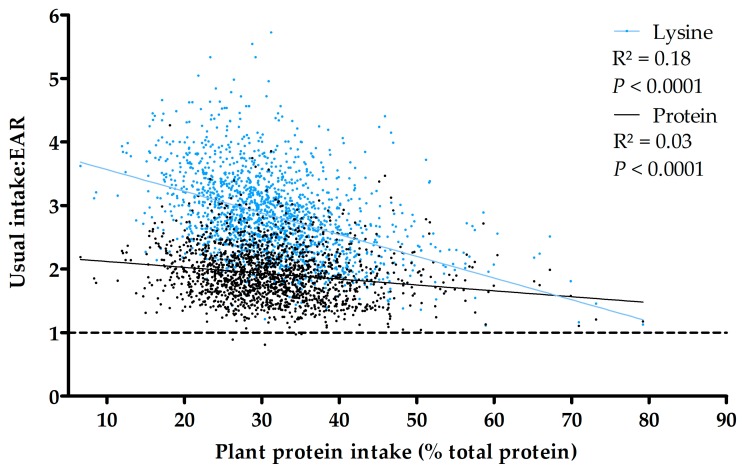
Association between protein and lysine intakes: EAR ratios and plant protein intake (% of total protein) in the diets. Points represent individuals of the French adult population (INCA2 study, *n* = 1678). The slopes of the linear regressions were significantly (*p* < 0.001) different from 0.

The intake:EAR ratios for protein and each IAA were negatively associated with the percentage of plant protein in the diets of French adults (*p* < 0.0001). However, the variability in intakes was poorly explained by the percentage of plant protein in the diets (*R*^2^ = 0.03 for protein and *R*^2^ < 0.1 for all IAA), except for lysine (*R*^2^ = 0.18) (Figure 3). The slope of the linear regression was low for the protein intake (−0.06 g/kg b.w./day for 10% more plant protein, i.e., −9% of the EAR) and higher for the lysine intake (−0.01 g/kg b.w./day for 10% more plant protein, i.e., −35% of the EAR). Results were still significant and similar, although numerically stronger, after adjustments for age, sex, BMI and energy intake as potential confounders (from β = −0.009 and −0.035 before adjustments to β = −0.018 and −0.046 for protein and lysine after adjustments, respectively).

Only the data on proteins and lysine are shown because the probability of an inadequacy of all other amino acids was lower than the probability of an inadequacy of proteins in all the simulations. 

According to the model adjusting for protein intake (Model P), simulations of a reduction in animal protein intake in favor of the same amount of plant protein led to an important increase in energy intake (~300 kcal per 10% increment in the percentage of plant protein). The simulation showed that the probability of a lysine inadequacy increased with the percentage of plant protein to reach 5% (3.96; 6.04) for a mean of 85% plant protein in the diets.

**Figure 4:**
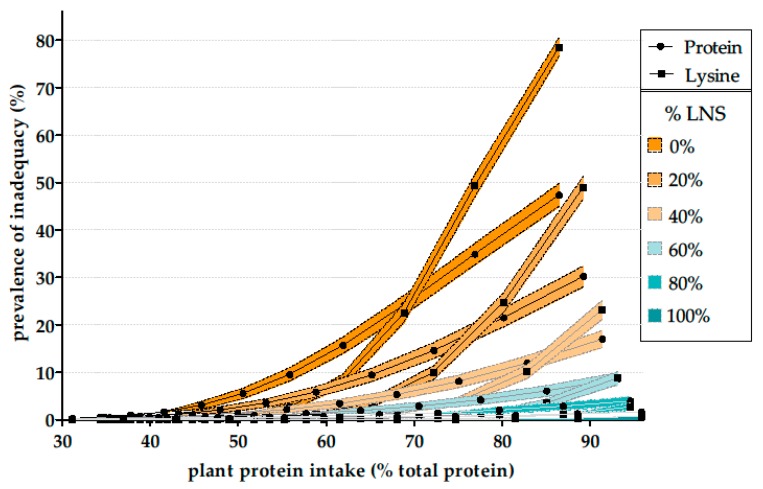
Prevalence of protein and lysine adequacy (% of the INCA2 study population, *n* = 1678) in simulations of a reduction in animal protein intake by gradually balancing it against the same amount of energy from a substituting combination composed of plant foods already consumed by individuals and a mixture of legumes, nuts and seeds. For example, the “40%” curves show the protein and lysine inadequacy when substituting animal protein by a combination of 40% of protein from legumes, nuts and seeds, and 60% of plant protein from foods already consumed by the individuals. The filled area represents the 95% confidence interval. LNS, Legumes, nuts and seeds.

According to the model adjusting for energy intake (Model A), simulations of a reduction in animal protein intake in favor of the same of energy from plant protein sources led to an increase in the probability of protein inadequacy which reached 5% (95% CI: 3.96; 6.04) and 50% (47.61; 52.39) for mean plant protein contents of 50% and 85% in the diets, respectively. Lysine inadequacy also increased to reach 5% (3.96; 6.04) when the mean plant protein content of the diets reached 58%. Lysine inadequacy became higher than protein inadequacy when plant protein accounted for >70% in the diets, to reach almost 80% (78.09; 81.91) with a mean plant protein content of 85% (Figure 4). 

According to the model substituting animal protein with legumes, nuts and seeds only (Model B), virtually the entire population had adequate protein and amino acid intake, at every level of plant protein in the diets. Results were similar according to the model that used as a substituting combination 80% legumes, nuts and seeds, and 20% of plant foods as already consumed (Model C_80_). The probability of protein inadequacy reached 5% (3.96; 6.04) when the mean plant protein contribution to total protein intake was >80%, 66% and 55% in models C_60_, C_40_ and C_20_, respectively. Lysine inadequacy became higher than protein inadequacy when the mean percentage of plant protein in the diets were >93%, 84%, 77% in models C_60_, C_40_ and C_20_ (Figure 4). Mean intake of legumes reach an average portion per day (150 g) for 50%, 53%, 60% and 75% of mean plant protein in models B, C_80_, C_60_ and C_40_, respectively.

## 4. Discussion

In this study we evaluated the adequacy of protein and amino acid intake in a western adult population and assessed their sensitivity to changing the plant:animal protein ratio. We found that in the current situation, protein and amino acid intake is adequate in virtually all adults, but an increase in the plant:animal protein ratio might primarily lead to an inadequacy of protein, and an inadequacy of lysine at the highest plant:animal protein ratios if keeping with the current pattern of plant protein intake. The introduction of a higher proportion of legumes, nuts and seeds when substituting for animal protein led to an adequate protein and lysine intake with higher plant:animal protein in the diet. 

### 4.1. Protein and Amino Acid Intakes and Adequacy Regarding Requirements

Using the most recent dietary intake data, our results showed that only 0.3% of the population had an inadequate protein intake and virtually all individuals had amino acid intakes higher than their requirements.

Protein intake and adequacy assessments produced similar results during the NHANES 2003–2004 study in the USA, except for women over 30 [13], and slightly higher results during the NHANES 2007–2010 study (3.8% of the population having inadequate intakes) [20]. In France, previous assessments generated results that were the same [14] or similar [37] to ours regarding protein intake and adequacy. A study assessing protein intake and adequacy worldwide revealed virtually no inadequacies in Eastern Europe and North America [38]. 

The mean amino acid intakes estimated were in line with those in a previous study in Japan [18] where only the proline intake was lower, a finding that could be explained by a higher consumption of dairy products in France [21,39]. Like the present study, this assessment did not produce any evidence of amino acid inadequacies. In the USA, amino acid intakes were assessed from the NHANES III (1988–1994) study [17] and the results were similar in men only, which can be explained by a low protein intake in women. In France, amino acid intake was assessed for lysine and methionine only and similar results were found [19] (see Table S2). 

It is necessary to underline a degree of uncertainty regarding how protein bioavailability have been taken into account in this study. Indeed, although the Digestible Indispensable Amino Acid Score (DIAAS) has been proposed as the most conceptually relevant approach to take account of bioavailability when estimating the quality of dietary protein, too few data are available on real ileal digestibility in humans and its use in the implementation of the DIAAS was not recommended until more data would become available. Furthermore, it has been acknowledged that using faecal digestibility can produce inaccurate results [1]. We therefore decided that the best option to account for differences in digestibility between plant and animal proteins in a mixed protein diet was to apply an average coefficient of 5% to lower plant protein intakes. Although some uncertainties are attached to estimating this coefficient, it is derived from the most precise estimates of the real ileal digestibility of a set of animal and plant proteins in humans [5]. It is uncertain whether this estimate will remain accurate in the coming years when new measurements on a wider variety of foods will be carried out as recommended in the FAO 2013 report [1]. The approach of assessing protein and amino acid adequacy separately bears some similarities with the amino acid scores concept. If the protein adequacy was adjusted for amino acid score, the protein adequacy would have been equal to the lysine adequacy from a mean plant protein content of 70% in the model A.

The Multiple Source Method used to assess usual protein and amino acids intake hypothesizes a statistical independence between the short-term records of food intake [32], whereas the seven days of records in INCA2 were on consecutive days. However, seven days of records clearly increase the precision of assessment as compared to two days. A Food Frequency Questionnaire was not required to assess usual intake for protein and amino acids, which are consumed every day. 

### 4.2. Foods Contributing to Protein and Amino Acid Intakes

Although cereals contain less protein than other food products (e.g., 15% protein as percentage of energy for cooked pasta, 50–80% for meat and 25–30% for legumes in this study), they contributed 21% to total protein intake, as they were widely consumed. However, cereals contain less lysine than other food products (e.g., 3 g/100 g of protein for cooked pasta, 9 g/100 g protein for red meat and 7 g/100 g protein for legumes). As a result, cereals accounted for only 9% of the total lysine intake.

The foods contributing to total, animal and plant protein intakes appeared to be similar to those in the NHANES 2007–2010 study [7], although a comparison could not be drawn directly because the foods identified in this latter study were not broken down into ingredients as in the present study. In another study on an US population in 1999–2002, the percentages of plant protein (34%) and eggs, pork, seafood intakes were similar, whereas the poultry intake was higher and beef and dairy intakes were lower than during the present study [40]. In Europe, the percentage of plant protein was found to be similar to our study, except in Italy [6] and, as in the present study, the cereals were the highest contributor to plant protein intake (from 45% to 70% depending on the country). In view of these similarities, it can be assumed that the French diet may be representative of a western diet with regards the levels and patterns of plant and animal proteins in the diet.

### 4.3. Association between Protein and Lysine Adequacy and Dietary Plant Protein Intake

There was a significant inverse association between the percentage of plant protein in the diet and the total protein intake, but the slope was low. Even with the highest levels of plant protein, intake was higher than the requirements in practically the entire study population. These results were in line with the U.S. report by the Dietary Guidelines Advisory Committee [12]. The extrapolation of regression lines for higher plant protein percentages did not suggest any potential inadequacy of protein intake. By contrast, the same extrapolation for lysine intake suggested that lysine adequacy could be of concern at very high plant protein intakes. However, because the range of plant protein intake in our population was relatively narrow (5th percentile, P5 = 19%; 95th percentile, P95 = 45%), an extrapolation to higher plant protein intake should be made with caution (Figure 2). 

Using a model adjusting for protein intake (Model P), we simulated that the prevalence of inadequate amino acid intakes in the population would reach 5%, with a mean plant protein content of 85% (P5 = 70%, P95 = 97%) in the diet, mostly because of lysine inadequacy (those of other amino acids always being lower than that of lysine). However, the simulated increase in energy intake was unrealistic (with a mean energy intake of ~4000 kcal for an 85% plant protein content). This could be ascribed to the high contribution of cereal foods to plant protein intake in the population. This result well illustrates the point that the plant protein foods consumed at present contain a low percentage of protein relative to energy, when compared with animal protein sources, as shown in the literature [41]. We considered this model to be unrealistic because it involves very large increases in energy intake while maintaining protein intake at a constant level. However, it can be pointed out that this is what the application of the protein leverage hypothesis [42] would imply in the context of such changes in protein sources.

Using a second model adjusting for energy intake (Model A), we simulated that above a mean 50% plant protein content in the diet (P5 = 38%, P95 = 64%), protein intake was inadequate, thus further illustrating the point that the plant-based foods consumed at present are too low in protein when compared to animal-based foods for a safe increase in plant:animal protein intake. Another important finding of this latter model was that up to a mean level of 70% (P5 = 56%, P95 = 80%) plant protein, the main issue was total protein intake and not the adequacy of lysine intake. The quality of the dietary protein consumed only became a critical parameter at plant protein intakes higher than a mean of 70%. That lysine was the most limiting of the individual amino acid could be clearly ascribed, again, to the importance of cereals to the plant protein intake of the population. However, a low protein:energy ratio appeared to be a more critical parameter than a low lysine:protein ratio. 

These models and simulations were based on the hypothesis that the reduction in animal protein intake was the same in each individual’s animal food items, and the increase in plant protein intake was also the same in each individual’s plant protein food items. Therefore, the conclusions of these models would only apply to the situation where people would not modify their plant protein intakes while consuming less animal protein. This may not be the case if the population is managing the pattern of protein intake during this transition. Vegetarians or vegans, who often tend to have a lower protein intake than omnivores [15,16,43], consume more greens and beans, more seaweeds and plant proteins than omnivores [15], and more legumes [6] or soy proteins [16]. By contrast, it is possible that a rapid change toward less animal protein in the more general population with less nutritional knowledge may cause changes to the plant:animal protein ratio with a similar pattern of plant protein sources, therefore resulting in protein inadequacy as simulated in model A. 

To go further in the analysis and use more realistic models than models P and A, we simulated the introduction of various proportions of legumes, nuts and seeds to substitute animal protein, as these foods have higher protein:energy ratios than cereals and are often advocated as the most valuable substitute for animal protein. These modellings (Models B, C_20_, C_40_, C_60_ and C_80_) showed that favoring legumes, nuts and seeds over the plant foods already consumed when substituting for animal protein allowed adequate protein and amino acids intakes for high proportions of plant protein in the diets. With high plant protein intake, securing protein and amino acids adequacy requires important changes in the pattern of plant protein sources as cereals and legumes, nuts and seeds, which may be viewed as an important obstacle to implement such a transition for the general population. This is not surprising since these protein models simulate up to a vegan diet for the entire population. However, conversely, and more relevant to the years to come, we could show that intermediary, yet important, transitions (i.e., resulting in 50 or 60% of plant protein) requires minor changes in the dietary pattern.

It should be noted that we restricted our study to the issue of protein and amino acid adequacy and did not consider the adequacy of other nutrients, which need to be considered in order to estimate the overall alignment with nutrient requirements of diets containing varying levels of plant proteins. In this regard, studies have shown that the diets of vegans or vegetarians have a higher nutritional quality than those of omnivores [15], with plant protein being associated with a higher overall nutrient adequacy [8,44]; the higher nutritional qualities of diets are estimated for individuals with high percentages of energy obtained from plant-based products [45,46]. The degree to which these relationships might vary with changes to the patterns of plant protein sources that seem to be necessary to ensure protein and amino acid adequacy when increasing the share of plant protein in the diet, remains unknown.

## 5. Conclusions

We conclude that if the population continues to follow the same plant protein food structure, which is low in protein, the adequacy of protein intake is not compromised until the average plant protein percentage rises from 30% to 50%. In this case, there is no primary question of lysine adequacy, but of protein in general. Under the same assumptions concerning the plant protein food pattern, there are no limitations specifically due to lysine with contribution of <70% of plant protein. Adequate protein and amino acids intake with high plant:animal protein ratios in the diet requires changes in the pattern of plant protein intake. Protein and lysine adequacy is efficiently secured when favoring legumes, nuts and seeds in the substitution, which is ascribed to their higher protein:energy ratios as compared to cereals.

Our findings call for further studies to simulate individual dietary modifications in a general population that might lead to a different balance of the plant:animal protein ratio, and to assess the nutritional quality of these changes using an overall evaluation of the nutrient adequacy of various diets.

## Figures and Tables

**Table 1:** Food groups for animal and plant proteins contributing to protein and lysine intake in the French adult population (INCA2 study) ^1^.

Food Contributing to Intake	% Protein Intake ^2^		% Lysine Intake ^2^	
	Men	Women	Men	Women
Animal	69.2 ± 8.4	68.5 ± 7.7	83.7 ± 5.9	82.5 ± 5.7
Meat	40.6 ± 13.6	34.7 ± 11.2	51 ± 15.3	43.5 ± 13.3
Red meat	19.4 ± 11.2	16.6 ± 9.2	23.7 ± 13.7	20.1 ± 11.2
Poultry	10.7 ± 10.4	9.3 ± 8	14.7 ± 13.7	12.8 ± 10.7
Game	0.4 ± 2.2	0.1 ± 1.1	0.5 ± 3	0.2 ± 1.5
Offal	0.8 ± 2.3	0.8 ± 2.2	0.9 ± 2.6	1 ± 2.5
Delicatessen	9.4 ± 6.1	7.8 ± 5.2	11.2 ± 7.6	9.4 ± 6.4
Fish	6.3 ± 6.6	8.2 ± 6.4	8.3 ± 8.6	10.7 ± 8.5
Dairy products	19 ± 9.1	21.4 ± 7.9	21.3 ± 10.9	24.3 ± 9.4
Milk	5.2 ± 5.7	6.6 ± 5.6	5.7 ± 6.7	7.2 ± 6.5
Yogurt	3.2 ± 4.1	4.9 ± 4.1	3.9 ± 4.9	5.9 ± 4.9
Cheese	10.2 ± 6.6	9.5 ± 5.7	11.4 ± 7.8	10.8 ± 6.8
Other dairy products	0.4 ± 0.3	0.5 ± 0.4	0.4 ± 0.3	0.4 ± 0.4
Eggs	3.3 ± 2.5	4.3 ± 2.7	3.1 ± 2.6	4 ± 2.9
Plant	30.8 ± 8.4	31.5 ± 7.7	16.3 ± 5.9	17.5 ± 5.7
Cereals	21.5 ± 7.4	20.2 ± 6.3	8.9 ± 4.3	8.6 ± 3.5
Potatoes	1.8 ± 1.2	1.9 ± 1.2	1.6 ± 1.1	1.8 ± 1.1
Fruit	1.6 ± 1.4	2.2 ± 1.9	1 ± 1.1	1.5 ± 1.5
Vegetables	2.4 ± 1.4	3.1 ± 1.5	2.3 ± 1.4	2.9 ± 1.4
Nuts and seeds	0.8 ± 1.5	0.8 ± 1.2	0.4 ± 0.7	0.4 ± 0.6
Legumes	1 ± 1.7	1.1 ± 1.9	1.1 ± 1.8	1.1 ± 2
Other plant products	1.2 ± 1	1.5 ± 1.1	0.6 ± 0.8	0.8 ± 0.9
Seasonings	0.5 ± 0.4	0.6 ± 0.7	0.4 ± 0.4	0.5 ± 0.6

^1^ INCA2, Individual and National Consumption Survey 2; *n* = 1678 (717 men and 961 women); ^2^ Mean ± Standard Deviation. The contributions of each food group to the intake of individual AA are shown Table S5.

## Supplementary Materials

The following are available online at www.mdpi.com/2072-6643/9/12/1333/s1, Method S1: French amino acid database, Table S1: Estimated average requirements of adults for protein and amino acids, from the 2002 Joint FAO/WHO/UNU Expert Consultation on Protein and Amino Acid Requirements in Human Nutrition, Table S2: Means and standard deviations of protein and amino acid intakes in the total population of both men and women (mg/kg b.w./day) compared with previous studies, Table S3: Means and standard deviations of protein and amino acid intakes stratified by gender and age (mg/kg b.w./day), Table S4: Means and standard deviations of protein and amino acid intakes stratified by gender and Body Mass Index (BMI) (mg/kg b.w./day), Table S5: Animal and plant food groups contributing to protein and amino acid intakes (%).
