# Supplementary material for: Protein Adequacy Is Primarily a Matter of Protein Quantity, Not Quality: Modeling an Increase in Plant:Animal Protein Ratio in French Adults
Source: Nutrients. 2017 Dec 8;9(12):1333. doi: 10.3390/nu9121333 (PMC5748783; doi:10.3390/nu9121333)
Supplement: Supplementary file 1 [file nutrients-09-01333-s001.zip › OSM.docx]

**Online supporting material**

**Method S1: French amino acid database**

An amino acid database was developed for the 1337 food items in the full repertoire of adults in the INCA2 study, using a method adapted from a study that described the development of an amino acid database in Japan by Suga et al. ([1](#_ENREF_1)).

***Sources of amino acid content***

The amino acid contents of different food items were collected from published French sources ([2](#_ENREF_2)), and international databases (USDA, Health Canada, the Standard Tables of Food Composition in Japan and the New Zealand Food Composition Database). These data came from amino acid analytical data obtained using automatic amino acid analyzers (with ion-exchange chromatography) or high performance liquid chromatography (HPLC). Each amino acid content was converted into mg/100 g of protein.

***Assignment with INCA2 food items***

To assign the amino acid contents of the foods analyzed to INCA2 food items, we used a 6-step procedure, as follows (Table 1):

*Step 1: Direct analytical data from French published sources*

Very few analyses had been performed on French foods using chromatographic methods to analyze 18 amino acids. Data from a study on the nutritional value of meat by the Centre d’Information des Viandes were used for most of the beef, veal, lamb, horse meat and offal food items (n=28) ([2](#_ENREF_2)).

*Step 2: Direct analytical data from international databases*

The amino acid compositions of foods obtained from four international databases were used to complete the French analyses ([3-6](#_ENREF_3)).

To determine the similarity of the foods, we used food item descriptors (taking account of the name of the product and cooking/process information), and the macronutrient content of foods (total fat, carbohydrates, protein, water and energy intake). A “similarity score” (S-score) was developed to assess the nutrient similarity between foods in the INCA2 survey and the food items found in international database:

*S-score = |Fat_i_ - Fat_INCA2_| + |Carb_i_ - Carb_INCA2_| + |Prot_i_ - Prot_INCA2_| + |Wat_i_ - Wat_INCA2_| + |(En_i_ - En_INCA2_)/5|*

i: food from international database, INCA2: food from the INCA2 study, Fat: total fat content (g/100g), Carb: total carbohydrate content (g/100g), Prot: total protein content (g/100g), Wat: total water content (g/100G), En: total energy (kcal/100g)

NB: The energy intake was divided by five as this ranges from P5 = 13kcal/100g to p95=511 kcal/100g, whereas other nutrients are expressed as a percentage.

Only foods with a similar name and cooking process, and an S-score <= 20 were assigned to INCA2 foods (n=435).

*Step 3: Data based on “similar” food items*

When no direct analytical data corresponded to INCA2 food items, assignments were made with “similar” food items. First of all (a), if data were available for a different form of the same food (e.g. cooked and not raw), we hypothesized that the amino acid profiles of the proteins were not modified by the cooking processes and we assigned data from a different form of the same food to the INCA2 food items (n = 189). Then (b), if data were unavailable for a food item but existed for similar species (e.g. food from different cuts of the same animal), we assigned the similar food to the INCA2 food item (n = 28).

*Step 4: Use of recipes to break down mixed food items*

Mixed INCA2 food items for which no data were found (e.g. chili con carne or lasagna) were broken down into ingredients using the INCA2 recipe table, and the food item amino acid content was calculated as a combination of the amino acid contents of its ingredients (n=409).

*Step 5: Non-analytical data from international databases*

When INCA2 food items with no corresponding data from previous steps had a corresponding food item from non-analytical data in international databases (e.g. mangos or guavas), the amino acid content of these data were used (n=28).

*Step 6: Assignment to 0 for foods containing no or very little protein*

The amino acid content of the remainder of the INCA2 food items was assigned to 0 as these foods contained no or very low protein contents (e.g. oil or alcoholic beverages). Those food items contributed less than 0.1% to the total protein intake in INCA2 (n=223).

*Table 1: Number of INCA2 food items assigned at each step of the procedure*

| Step | Number of INCA2 food items |
| --- | --- |
| 1 | 28 |
| 2 | 435 |
| 3 | 217 |
| 4 | 409 |
| 5 | 28 |
| 6 | 220 |

***Calculation of the amino acid contents of foods***

The contents in amino acids per 100g of food were calculated as follows:

*[Amino acid]_INCA2_ (mg/100g food) = [Amino acid]_other_ (mg/100g protein) / 100 * [Protein]_INCA2_ (g/100g food)*

When an INCA2 food item corresponded to more than one food item from other sources, the amino acid value used was the mean of the corresponding values for the food item, weighted by the S-Score.

***Assessment of database quality***

Five grades (A+, A, B, C, D) were assigned to each INCA2 food item in order to define the quality of the value (Table 2). The grade rose if:

- the data was not from French analyses,
- it was not the same form of the food,
- a biologically similar food was used,
- a recipe was used to break down the food into ingredients,
- a non-analytical value was used,
- the value was estimated to be 0,
- the S-score was >10.

The grade fell if:

- more than one food item from the international databases corresponded to the INCA2 food item, and the mean coefficient of variation of the different amino acid values in these items was <10%.

*Table 2: Quality of the amino acid data for INCA2 food items*

| Quality of the INCA2 food item | Number of INCA2 food items |
| --- | --- |
| A+ | 42 |
| A | 255 |
| B | 908 |
| C | 116 |
| D | 16 |

***Validation of the database***

FAO/INFOODS guideline checks were used to validate the database ([7](#_ENREF_7)).

- The checks on food identification were completed because the data came from international databases and accurate publications. Moreover, other foods were assigned to INCA2 food categories according to the similarity of precise names and macronutrient contents. However, the assignments of INCA2 food items to different forms of the same food or foods of similar species did not comply with these guidelines. The scoring system was applied to address this issue.
- The checks on components were not necessary as only amino acid values per 100g of protein were used. To assess the validity of using different sources in the database, the coefficients of variation of the amino acid contents for each food group of the INCA2 nomenclature were calculated. It appeared that among the 40% most homogeneous groups (meat, dairy products, eggs, fish, rice, wheat, bread, pasta, etc.), the mean CV values for amino acids were <10%. Less homogeneous groups which accounted for 29% of the groups (legumes, sandwiches, cakes, offal, potatoes, vegetables, etc.) had mean CV values of between 10% and 20%. Finally, the 31% least homogeneous groups (vegetables dishes, vegetables, fruits, juices, seasonings, etc.) had mean CV values >20. This suggested that even though the data came from a combination of different sources, foods of the same origin had similar amino acid profiles.
- The checks on recipes were verified as the recipes came from the Anses INCA2 recipe table.
- The checks on data documentation were not always complied with in the international databases. Indeed, and particularly for amino acids, the number of foods analyzed and their standard deviations were rarely complete. However, given the reputation of the international databases it can be assumed that the data were reliable.

**Table S1: Estimated average requirements of adults for protein and amino acids, from the 2002 Joint FAO/WHO/UNU Expert Consultation on Protein and Amino Acid Requirements in Human Nutrition (**[**8**](#_ENREF_8)**)**

| Nutrient | EAR (mg/kg b.w./d) |
| --- | --- |
| Protein | 660 |
| Valine | 26 |
| Tryptophan | 4 |
| Threonine | 15 |
| Sulphur | 15 |
| Lysine | 30 |
| Leucine | 39 |
| Isoleucine | 20 |
| Histidine | 10 |
| Aromatic | 25 |

**Table S2: Means and standard deviations of protein and amino acid intakes in the total population of both men and women (mg/kg b.w./d) compared with previous studies**

| Nutrient | INCA2 (2006 – 2007) | | | Suga et al. ([1](#_ENREF_1)) | | Martin et al. ([9](#_ENREF_9))^1^ | Trumbo et al. ([10](#_ENREF_10))^2^ | |
| --- | --- | --- | --- | --- | --- | --- | --- | --- |
|  | Total | Men | Women | Men | Women | Total | Men | Women |
| Aspartic acid | 111 (±29) | 114 (±30) | 109 (±27) | 109 | 114 | - | 114 | 93 |
| Alanine | 62 (±17) | 64 (±18) | 59 (±16) | 60 | 61 | - | 64 | 52 |
| Arginine | 69 (±19) | 72 (±20) | 66 (±17) | 70 | 71 | - | 74 | 60 |
| Cysteine | 18 (±5) | 19 (±5) | 17 (±4) | 18 | 19 | - | 17 | 14 |
| Glutamic acid | 259 (±64) | 270 (±68) | 250 (±59) | 212 | 226 | - | 259 | 216 |
| Glycine | 53 (±15) | 56 (±16) | 51 (±14) | 55 | 55 | - | 57 | 46 |
| Histidine | 38 (±10) | 39 (±11) | 36 (±9) | 41 | 41 | - | 38 | 31 |
| Isoleucine | 57 (±14) | 59 (±16) | 55 (±13) | 51 | 53 | - | 61 | 50 |
| Leucine | 102 (±26) | 106 (±28) | 99 (±24) | 91 | 95 | - | 104 | 86 |
| Lysine | 88 (±24) | 91 (±26) | 85 (±23) | 77 | 80 | 87 | 92 | 75 |
| Methionine | 31 (±8) | 32 (±9) | 30 (±8) | 28 | 28 | 30 | 31 | 25 |
| Phenylalanine | 58 (±14) | 60 (±15) | 56 (±13) | 53 | 55 | - | 58 | 48 |
| Proline | 92 (±23) | 96 (±25) | 89 (±22) | 66 | 72 | - | 87 | 72 |
| Serine | 60 (±15) | 62 (±16) | 59 (±14) | 55 | 58 | - | 60 | 50 |
| Threonine | 52 (±14) | 54 (±15) | 50 (±13) | 47 | 49 | - | 52 | 43 |
| Tryptophan | 15 (±4) | 15 (±4) | 14 (±3) | 14 | 15 | - | 16 | 13 |
| Tyrosine | 46 (±12) | 48 (±13) | 45 (±11) | 41 | 43 | - | 48 | 39 |
| Valine | 66 (±17) | 68 (±18) | 64 (±16) | 60 | 63 | - | 52 | 56 |
| Protein | 1289 (±320) | 1336 (±341) | 1247 (±300) | - | - | - | 1274 | 1056 |

^1^ Intakes (mg/d) were converted to mg/kg b.w./day considering a body weight of 70kg

^2^ Intakes (mg/d) were converted to mg/kg b.w./day considering a body weight of 60kg for women and 75kg for men

**Table S3: Means and standard deviations of protein and amino acid intakes stratified by gender and age (mg/kg b.w./d)**

|  | Men | | | | Women | | | |
| --- | --- | --- | --- | --- | --- | --- | --- | --- |
|  | **18 - 24 (n=67)** | **25 - 34 (n=114)** | **35 - 49 (n=249)** | **50 - 65 (n=287)** | **18 - 24 (n=95)** | **25 - 34 (n=204)** | **35 - 49 (n=349)** | **50 - 65 (n=313)** |
| Aspartic acid | 120 (± 40) | 114 (± 39) | 114 (± 29) | 112 (± 25) | 108 (± 34) | 108 (± 26) | 111 (± 29) | 108 (± 25) |
| Alanine | 69 (± 24) | 65 (± 24) | 64 (± 16) | 63 (± 14) | 60 (± 20) | 59 (± 15) | 60 (± 16) | 58 (± 14) |
| Arginine | 76 (± 27) | 72 (± 25) | 72 (± 19) | 70 (± 16) | 67 (± 23) | 66 (± 17) | 68 (± 18) | 64 (± 15) |
| Cysteine | 20 (± 7) | 19 (± 6) | 19 (± 5) | 18 (± 4) | 18 (± 6) | 18 (± 4) | 18 (± 5) | 17 (± 4) |
| Glutamic acid | 286 (± 88) | 273 (± 82) | 270 (± 64) | 262 (± 58) | 252 (± 73) | 253 (± 57) | 255 (± 62) | 239 (± 53) |
| Glycine | 60 (± 21) | 56 (± 20) | 56 (± 15) | 55 (± 12) | 52 (± 18) | 51 (± 13) | 52 (± 14) | 50 (± 12) |
| Histidine | 41 (± 15) | 40 (± 14) | 39 (± 10) | 38 (± 9) | 36 (± 11) | 36 (± 9) | 37 (± 10) | 35 (± 8) |
| Isoleucine | 62 (± 20) | 60 (± 19) | 59 (± 14) | 57 (± 13) | 55 (± 17) | 55 (± 13) | 56 (± 14) | 53 (± 12) |
| Leucine | 112 (± 36) | 108 (± 36) | 106 (± 25) | 103 (± 24) | 99 (± 30) | 99 (± 24) | 101 (± 25) | 96 (± 21) |
| Lysine | 96 (± 35) | 92 (± 33) | 90 (± 24) | 89 (± 22) | 84 (± 28) | 84 (± 22) | 86 (± 23) | 83 (± 20) |
| Methionine | 35 (± 12) | 33 (± 12) | 32 (± 8) | 31 (± 7) | 30 (± 10) | 30 (± 8) | 31 (± 8) | 29 (± 7) |
| Phenylalanine | 64 (± 19) | 61 (± 19) | 60 (± 14) | 59 (± 13) | 57 (± 16) | 57 (± 13) | 58 (± 14) | 55 (± 12) |
| Proline | 101 (± 32) | 98 (± 28) | 96 (± 24) | 92 (± 22) | 90 (± 25) | 91 (± 21) | 92 (± 23) | 84 (± 19) |
| Serine | 66 (± 20) | 63 (± 19) | 62 (± 15) | 60 (± 13) | 59 (± 17) | 59 (± 13) | 60 (± 15) | 57 (± 12) |
| Threonine | 58 (± 19) | 55 (± 19) | 54 (± 13) | 52 (± 12) | 51 (± 16) | 51 (± 12) | 52 (± 13) | 49 (± 11) |
| Tryptophan | 16 (± 5) | 15 (± 5) | 15 (± 4) | 15 (± 3) | 14 (± 4) | 14 (± 3) | 15 (± 4) | 14 (± 3) |
| Tyrosine | 50 (± 16) | 49 (± 16) | 48 (± 12) | 47 (± 11) | 45 (± 13) | 45 (± 11) | 46 (± 12) | 44 (± 10) |
| Valine | 72 (± 22) | 69 (± 22) | 68 (± 16) | 66 (± 15) | 65 (± 19) | 64 (± 15) | 66 (± 16) | 62 (± 14) |
| Protein | 1410 (± 441) | 1352 (± 428) | 1336 (± 315) | 1302 (± 285) | 1251 (± 372) | 1251 (± 289) | 1277 (± 312) | 1208 (± 264) |

**Table S4: Means and standard deviations of protein and amino acid intakes stratified by gender and Body Mass Index (BMI) (mg/kg b.w./d)**

|  | Men | | | Women | | |
| --- | --- | --- | --- | --- | --- | --- |
|  | Normal weight (n=401) | Pre-obesity (n=244) | Obesity (n=72) | Normal weight (n=713) | Pre-obesity (n=176) | Obesity (n=72) |
| Aspartic acid | 120 (+/- 33) | 110 (+/- 24) | 94 (+/- 21) | 113 (+/- 28) | 100 (+/- 23) | 85 (+/- 19) |
| Alanine | 68 (+/- 20) | 62 (+/- 14) | 53 (+/- 12) | 61 (+/- 16) | 54 (+/- 12) | 46 (+/- 11) |
| Arginine | 76 (+/- 22) | 69 (+/- 16) | 59 (+/- 13) | 69 (+/- 18) | 61 (+/- 14) | 51 (+/- 12) |
| Cysteine | 20 (+/- 5) | 18 (+/- 4) | 15 (+/- 3) | 18 (+/- 4) | 16 (+/- 3) | 13 (+/- 3) |
| Glutamic acid | 285 (+/- 71) | 259 (+/- 57) | 216 (+/- 41) | 261 (+/- 60) | 222 (+/- 42) | 191 (+/- 38) |
| Glycine | 59 (+/- 17) | 54 (+/- 12) | 46 (+/- 11) | 53 (+/- 14) | 47 (+/- 11) | 39 (+/- 10) |
| Histidine | 41 (+/- 12) | 38 (+/- 9) | 32 (+/- 7) | 37 (+/- 10) | 33 (+/- 7) | 28 (+/- 7) |
| Isoleucine | 62 (+/- 17) | 56 (+/- 13) | 48 (+/- 9) | 57 (+/- 14) | 50 (+/- 10) | 42 (+/- 9) |
| Leucine | 112 (+/- 30) | 102 (+/- 23) | 86 (+/- 17) | 103 (+/- 25) | 90 (+/- 18) | 76 (+/- 17) |
| Lysine | 95 (+/- 29) | 87 (+/- 22) | 75 (+/- 17) | 87 (+/- 23) | 78 (+/- 18) | 66 (+/- 17) |
| Methionine | 34 (+/- 10) | 31 (+/- 7) | 26 (+/- 6) | 31 (+/- 8) | 27 (+/- 6) | 23 (+/- 6) |
| Phenylalanine | 63 (+/- 16) | 58 (+/- 13) | 49 (+/- 10) | 59 (+/- 14) | 51 (+/- 10) | 43 (+/- 9) |
| Proline | 101 (+/- 26) | 91 (+/- 22) | 76 (+/- 15) | 93 (+/- 22) | 79 (+/- 15) | 67 (+/- 12) |
| Serine | 65 (+/- 17) | 59 (+/- 13) | 50 (+/- 10) | 61 (+/- 14) | 53 (+/- 10) | 45 (+/- 9) |
| Threonine | 57 (+/- 16) | 52 (+/- 12) | 44 (+/- 9) | 52 (+/- 13) | 46 (+/- 10) | 39 (+/- 9) |
| Tryptophan | 16 (+/- 4) | 15 (+/- 3) | 12 (+/- 2) | 15 (+/- 4) | 13 (+/- 3) | 11 (+/- 2) |
| Tyrosine | 51 (+/- 14) | 47 (+/- 11) | 39 (+/- 8) | 47 (+/- 11) | 41 (+/- 8) | 35 (+/- 8) |
| Valine | 72 (+/- 19) | 65 (+/- 15) | 55 (+/- 11) | 67 (+/- 16) | 58 (+/- 12) | 50 (+/- 10) |
| Protein | 1407 (+/- 363) | 1283 (+/- 277) | 1086 (+/- 212) | 1297 (+/- 305) | 1126 (+/- 224) | 960 (+/- 204) |

**Table S5: Animal and plant food groups contributing to protein and amino acid intakes (%)**

| **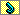Nutrient** | **Aspartic acid** | | **Alanine** | | **Arginine** | | **Cysteine** | | **Glutamic acid** | | **Glycine** | | **Histidine** | | **Isoleucine** | | **Leucine** | | **Lysine** | |
| --- | --- | --- | --- | --- | --- | --- | --- | --- | --- | --- | --- | --- | --- | --- | --- | --- | --- | --- | --- | --- |
|  | Men (n=717) | Women (n=961) | Men (n=717) | Women (n=961) | Men (n=717) | Women (n=961) | Men (n=717) | Women (n=961) | Men (n=717) | Women (n=961) | Men (n=717) | Women (n=961) | Men (n=717) | Women (n=961) | Men (n=717) | Women (n=961) | Men (n=717) | Women (n=961) | Men (n=717) | Women (n=961) |
| **Animal** | 72.2 ± 8.2 | 69.7 ± 7.9 | 76.3 ± 7.4 | 75 ± 7 | 73.8 ± 8.4 | 72 ± 8 | 62.6 ± 10 | 61.9 ± 8.9 | 61.2 ± 9.7 | 61.2 ± 8.8 | 73.2 ± 8.5 | 71.9 ± 8.1 | 76.1 ± 7.6 | 75.4 ± 7.2 | 74.8 ± 7.6 | 74.2 ± 7 | 74.1 ± 7.7 | 73.7 ± 7.1 | 83.7 ± 5.9 | 82.5 ± 5.7 |
| ***Meat*** | 44.3 ± 14.2 | 37.1 ± 11.9 | 50.7 ± 14.6 | 44 ± 12.8 | 49.6 ± 14.6 | 43 ± 12.7 | 41.3 ± 13.8 | 36.1 ± 11.8 | 33.7 ± 12.5 | 28.7 ± 10 | 51.2 ± 14.4 | 44.8 ± 12.8 | 47.7 ± 14.5 | 41.8 ± 12.6 | 43.1 ± 14.2 | 36.7 ± 11.9 | 42.5 ± 14.2 | 36.3 ± 11.7 | 51 ± 15.3 | 43.5 ± 13.3 |
| Red meat | 21.1 ± 12.1 | 17.6 ± 9.8 | 24.5 ± 13.7 | 21.3 ± 11.4 | 23.8 ± 13.4 | 20.6 ± 11.1 | 22.1 ± 12.8 | 19.3 ± 10.6 | 16.1 ± 9.5 | 13.7 ± 7.8 | 24.7 ± 13.7 | 21.7 ± 11.6 | 22.6 ± 12.9 | 19.5 ± 10.7 | 20.6 ± 12 | 17.6 ± 9.8 | 20.3 ± 11.8 | 17.3 ± 9.7 | 23.7 ± 13.7 | 20.1 ± 11.2 |
| Poultry | 12 ± 11.5 | 10.2 ± 8.7 | 13.1 ± 12.3 | 11.6 ± 9.8 | 13.3 ± 12.4 | 11.7 ± 9.9 | 8.8 ± 8.7 | 7.7 ± 6.8 | 8.8 ± 8.8 | 7.6 ± 6.8 | 12.2 ± 11.5 | 11 ± 9.4 | 12.6 ± 12.2 | 11.4 ± 9.8 | 11.5 ± 11.2 | 10 ± 8.7 | 11.3 ± 11 | 9.8 ± 8.5 | 14.7 ± 13.7 | 12.8 ± 10.7 |
| Game | 0.4 ± 2.5 | 0.2 ± 1.1 | 0.4 ± 2.7 | 0.2 ± 1.4 | 0.4 ± 2.6 | 0.2 ± 1.3 | 0.3 ± 1.9 | 0.1 ± 1 | 0.3 ± 1.8 | 0.1 ± 0.9 | 0.4 ± 2.5 | 0.2 ± 1.3 | 0.5 ± 3 | 0.2 ± 1.6 | 0.4 ± 2.5 | 0.2 ± 1.2 | 0.4 ± 2.3 | 0.2 ± 1.1 | 0.5 ± 3 | 0.2 ± 1.5 |
| Offal | 0.8 ± 2.5 | 0.9 ± 2.4 | 0.9 ± 2.8 | 1 ± 2.7 | 0.9 ± 2.8 | 1 ± 2.7 | 1.3 ± 4.1 | 1.6 ± 4.2 | 0.5 ± 1.7 | 0.6 ± 1.5 | 1.1 ± 3.3 | 1.2 ± 3.1 | 0.7 ± 2.2 | 0.8 ± 2.2 | 0.8 ± 2.3 | 0.9 ± 2.3 | 0.8 ± 2.5 | 0.9 ± 2.5 | 0.9 ± 2.6 | 1 ± 2.5 |
| Delicatessen | 10 ± 6.7 | 8.2 ± 5.6 | 11.6 ± 7.7 | 9.9 ± 6.6 | 11.2 ± 7.4 | 9.4 ± 6.3 | 8.9 ± 5.8 | 7.4 ± 4.9 | 8 ± 5.1 | 6.6 ± 4.3 | 12.8 ± 8.6 | 10.8 ± 7.3 | 11.3 ± 7.3 | 9.8 ± 6.4 | 9.8 ± 6.4 | 8.1 ± 5.4 | 9.6 ± 6.3 | 8.1 ± 5.4 | 11.2 ± 7.6 | 9.4 ± 6.4 |
| ***Fish*** | 7.5 ± 7.6 | 9.4 ± 7.3 | 8.3 ± 8.5 | 10.7 ± 8.3 | 7.6 ± 7.7 | 10 ± 7.7 | 5.4 ± 5.7 | 7 ± 5.5 | 5 ± 5.3 | 6.5 ± 5.2 | 8.3 ± 8.5 | 11.1 ± 8.9 | 6.6 ± 7.5 | 8.4 ± 7.4 | 6.7 ± 7.1 | 8.6 ± 6.8 | 6.6 ± 6.9 | 8.5 ± 6.7 | 8.3 ± 8.6 | 10.7 ± 8.5 |
| ***Dairy products*** | 16.9 ± 8.6 | 18.8 ± 7.4 | 13.9 ± 7.4 | 15.7 ± 6.4 | 13.1 ± 6.9 | 14.5 ± 5.9 | 11.2 ± 5.8 | 12.7 ± 5.1 | 19.6 ± 9.1 | 22.2 ± 8 | 10.9 ± 6.1 | 12.3 ± 5.2 | 19 ± 9.5 | 21.6 ± 8.3 | 21.4 ± 10.1 | 24.3 ± 8.9 | 21.6 ± 10.2 | 24.5 ± 8.9 | 21.3 ± 10.9 | 24.3 ± 9.4 |
| Milk | 5.1 ± 5.7 | 6.3 ± 5.4 | 3.9 ± 4.4 | 4.9 ± 4.2 | 3.4 ± 3.7 | 4.3 ± 3.4 | 3.3 ± 3.3 | 4.1 ± 3.1 | 5.5 ± 6 | 7 ± 5.9 | 3 ± 3.3 | 3.8 ± 3.1 | 5.1 ± 5.8 | 6.4 ± 5.6 | 5.7 ± 6.3 | 7.2 ± 6.2 | 5.8 ± 6.4 | 7.3 ± 6.3 | 5.7 ± 6.7 | 7.2 ± 6.5 |
| Yogurts | 2.9 ± 3.8 | 4.3 ± 3.6 | 2.4 ± 3.2 | 3.7 ± 3.2 | 2.1 ± 2.8 | 3.2 ± 2.8 | 1.7 ± 2.3 | 2.7 ± 2.5 | 3.2 ± 4.1 | 4.9 ± 4.2 | 1.9 ± 2.5 | 2.8 ± 2.4 | 3 ± 3.8 | 4.5 ± 3.9 | 3.9 ± 4.9 | 5.9 ± 5 | 3.8 ± 4.7 | 5.7 ± 4.9 | 3.9 ± 4.9 | 5.9 ± 4.9 |
| Cheese | 8.5 ± 5.8 | 7.8 ± 4.8 | 7.2 ± 4.9 | 6.8 ± 4.3 | 7.2 ± 4.9 | 6.7 ± 4.2 | 5.9 ± 4.1 | 5.5 ± 3.4 | 10.4 ± 6.6 | 9.7 ± 5.7 | 5.8 ± 4.1 | 5.3 ± 3.4 | 10.6 ± 7 | 10.1 ± 6.2 | 11.3 ± 7.4 | 10.6 ± 6.4 | 11.6 ± 7.6 | 10.9 ± 6.6 | 11.4 ± 7.8 | 10.8 ± 6.8 |
| Other | 0.4 ± 0.3 | 0.4 ± 0.4 | 0.3 ± 0.3 | 0.4 ± 0.3 | 0.3 ± 0.2 | 0.4 ± 0.3 | 0.3 ± 0.3 | 0.4 ± 0.3 | 0.4 ± 0.4 | 0.5 ± 0.4 | 0.3 ± 0.2 | 0.3 ± 0.3 | 0.3 ± 0.3 | 0.4 ± 0.3 | 0.4 ± 0.4 | 0.5 ± 0.5 | 0.4 ± 0.4 | 0.5 ± 0.4 | 0.4 ± 0.3 | 0.4 ± 0.4 |
| ***Eggs*** | 3.5 ± 2.9 | 4.4 ± 3 | 3.5 ± 3 | 4.6 ± 3.2 | 3.5 ± 2.9 | 4.6 ± 3.2 | 4.6 ± 3.7 | 6.1 ± 4.1 | 2.9 ± 2.1 | 3.8 ± 2.2 | 2.7 ± 2.2 | 3.6 ± 2.4 | 2.8 ± 2.2 | 3.7 ± 2.5 | 3.6 ± 2.9 | 4.6 ± 3.1 | 3.4 ± 2.7 | 4.4 ± 2.9 | 3.1 ± 2.6 | 4 ± 2.9 |
| **Plant** | 27.8 ± 8.2 | 30.3 ± 7.9 | 23.7 ± 7.4 | 25 ± 7 | 26.2 ± 8.4 | 28 ± 8 | 37.4 ± 10 | 38.1 ± 8.9 | 38.8 ± 9.7 | 38.8 ± 8.8 | 26.8 ± 8.5 | 28.1 ± 8.1 | 23.9 ± 7.6 | 24.6 ± 7.2 | 25.2 ± 7.6 | 25.8 ± 7 | 25.9 ± 7.7 | 26.3 ± 7.1 | 16.3 ± 5.9 | 17.5 ± 5.7 |
| Cereals | 12.8 ± 5.1 | 12 ± 4.1 | 15.3 ± 5.9 | 14.6 ± 4.9 | 15.7 ± 6.1 | 15.2 ± 5 | 29.3 ± 9.4 | 27.8 ± 7.8 | 30.6 ± 9.5 | 28.7 ± 8 | 18.6 ± 7 | 17.9 ± 6 | 16.8 ± 6.4 | 16 ± 5.5 | 17.7 ± 6.5 | 16.7 ± 5.5 | 18.6 ± 6.8 | 17.6 ± 5.7 | 8.9 ± 4.3 | 8.6 ± 3.5 |
| Potatoes | 4.2 ± 2.9 | 4.4 ± 2.8 | 1.4 ± 1 | 1.6 ± 1 | 1.8 ± 1.3 | 2 ± 1.3 | 1.8 ± 1.2 | 2 ± 1.3 | 1.7 ± 1.2 | 1.9 ± 1.3 | 1.4 ± 1 | 1.7 ± 1.1 | 1.3 ± 0.9 | 1.4 ± 0.9 | 1.6 ± 1.1 | 1.7 ± 1.1 | 1.4 ± 0.9 | 1.5 ± 0.9 | 1.6 ± 1.1 | 1.8 ± 1.1 |
| Fruit | 3.7 ± 4.2 | 5.5 ± 4.9 | 1.5 ± 1.5 | 2.2 ± 1.9 | 2.2 ± 2.3 | 3.1 ± 2.9 | 1.1 ± 1.2 | 1.7 ± 1.5 | 0.9 ± 1 | 1.4 ± 1.5 | 1.2 ± 1.3 | 1.8 ± 1.8 | 1.2 ± 1.3 | 1.6 ± 1.7 | 0.8 ± 1 | 1.3 ± 1.5 | 0.8 ± 0.9 | 1.3 ± 1.4 | 1 ± 1.1 | 1.5 ± 1.5 |
| Vegetables | 3.5 ± 2.1 | 4.5 ± 2.2 | 2.8 ± 1.7 | 3.6 ± 1.8 | 2.7 ± 1.8 | 3.4 ± 1.7 | 2.3 ± 1.3 | 3.1 ± 1.5 | 2.5 ± 1.4 | 3.3 ± 1.5 | 2.4 ± 1.5 | 3.2 ± 1.6 | 2 ± 1.2 | 2.5 ± 1.2 | 2.3 ± 1.3 | 2.9 ± 1.4 | 2.2 ± 1.3 | 2.7 ± 1.3 | 2.3 ± 1.4 | 2.9 ± 1.4 |
| Nuts and seeds | 0.9 ± 1.7 | 0.8 ± 1.2 | 0.7 ± 1.2 | 0.6 ± 1 | 1.3 ± 2.5 | 1.1 ± 1.8 | 0.9 ± 1.5 | 0.9 ± 1.4 | 0.9 ± 1.6 | 0.9 ± 1.4 | 0.9 ± 1.8 | 0.9 ± 1.4 | 0.7 ± 1.2 | 0.7 ± 1 | 0.7 ± 1.2 | 0.7 ± 1 | 0.7 ± 1.2 | 0.7 ± 1 | 0.4 ± 0.7 | 0.4 ± 0.6 |
| Legumes | 1.4 ± 2.4 | 1.4 ± 2.5 | 1 ± 1.7 | 1 ± 1.8 | 1.4 ± 2.4 | 1.5 ± 2.7 | 0.8 ± 1.3 | 0.8 ± 1.3 | 0.9 ± 1.5 | 0.9 ± 1.6 | 1.1 ± 1.8 | 1.1 ± 2 | 1 ± 1.7 | 1 ± 1.8 | 1.1 ± 1.8 | 1.1 ± 1.9 | 1.1 ± 1.8 | 1.1 ± 1.8 | 1.1 ± 1.8 | 1.1 ± 2 |
| Other | 0.8 ± 1 | 1 ± 1 | 0.6 ± 0.8 | 0.9 ± 0.9 | 0.7 ± 0.9 | 0.9 ± 1 | 0.8 ± 1 | 1 ± 1.1 | 0.8 ± 0.9 | 1 ± 1 | 0.6 ± 0.8 | 0.8 ± 0.9 | 0.7 ± 0.8 | 0.9 ± 0.9 | 0.7 ± 0.9 | 1 ± 1 | 0.7 ± 0.9 | 1 ± 1 | 0.6 ± 0.8 | 0.8 ± 0.9 |
| Seasonings | 0.6 ± 0.6 | 0.8 ± 1 | 0.4 ± 0.4 | 0.6 ± 0.6 | 0.5 ± 0.4 | 0.7 ± 0.7 | 0.5 ± 0.5 | 0.7 ± 0.7 | 0.6 ± 0.6 | 0.8 ± 0.9 | 0.5 ± 0.5 | 0.7 ± 0.7 | 0.4 ± 0.4 | 0.5 ± 0.5 | 0.4 ± 0.4 | 0.5 ± 0.5 | 0.4 ± 0.4 | 0.5 ± 0.5 | 0.4 ± 0.4 | 0.5 ± 0.6 |

**Table S5 (continued): Animal and plant food groups contributing to protein and amino acid intakes (%)**

| **Nutrient** | **Methionine** | | **Phenylalanine** | | **Proline** | | **Serine** | | **Threonine** | | **Tryptophan** | | **Tyrosine** | | **Valine** | | **Protein** | |  |  |
| --- | --- | --- | --- | --- | --- | --- | --- | --- | --- | --- | --- | --- | --- | --- | --- | --- | --- | --- | --- | --- |
|  | Men (n=717) | Women (n=961) | Men (n=717) | Women (n=961) | Men (n=717) | Women (n=961) | Men (n=717) | Women (n=961) | Men (n=717) | Women (n=961) | Men (n=717) | Women (n=961) | Men (n=717) | Women (n=961) | Men (n=717) | Women (n=961) | Men (n=717) | Women (n=961) |  |  |
| **Animal** | 80.2 ± 6.7 | 79.6 ± 6.1 | 69.2 ± 8.5 | 68.9 ± 7.8 | 62.1 ± 9.6 | 62.8 ± 8.6 | 70.1 ± 8.1 | 69.6 ± 7.4 | 75.8 ± 7.5 | 74.7 ± 7.1 | 70.6 ± 8.4 | 69.8 ± 7.5 | 75 ± 7.6 | 74.8 ± 7 | 73.8 ± 7.5 | 73.3 ± 6.9 | 69.2 ± 8.4 | 68.5 ± 7.7 |  |  |
| ***Meat*** | 48.9 ± 15.1 | 42.3 ± 12.9 | 38.4 ± 13.4 | 32.7 ± 10.9 | 29 ± 11.5 | 24.6 ± 8.9 | 37.6 ± 13.4 | 31.6 ± 10.7 | 45.8 ± 14.5 | 39.1 ± 12.2 | 38.9 ± 13.8 | 32.9 ± 11.3 | 39.8 ± 14 | 33.8 ± 11.5 | 40.8 ± 13.9 | 34.6 ± 11.4 | 40.6 ± 13.6 | 34.7 ± 11.2 |  |  |
| Red meat | 25.5 ± 14.4 | 22 ± 11.8 | 18.3 ± 10.7 | 15.5 ± 8.8 | 14.1 ± 8.7 | 12 ± 6.9 | 18 ± 10.6 | 15.1 ± 8.5 | 22.3 ± 12.8 | 19.1 ± 10.4 | 16.4 ± 9.9 | 13.7 ± 8.2 | 18.8 ± 11.2 | 15.9 ± 9.1 | 19.5 ± 11.4 | 16.5 ± 9.3 | 19.4 ± 11.2 | 16.6 ± 9.2 |  |  |
| Poultry | 12.3 ± 11.7 | 10.8 ± 9.2 | 9.9 ± 9.8 | 8.6 ± 7.5 | 6.5 ± 6.7 | 5.6 ± 5.1 | 9.7 ± 9.6 | 8.3 ± 7.3 | 12.1 ± 11.5 | 10.5 ± 9 | 11.9 ± 11.5 | 10.2 ± 8.8 | 11 ± 10.7 | 9.5 ± 8.3 | 10.5 ± 10.4 | 9 ± 8 | 10.7 ± 10.4 | 9.3 ± 8 |  |  |
| Game | 0.4 ± 2.4 | 0.2 ± 1.2 | 0.3 ± 2 | 0.1 ± 1 | 0.2 ± 1.3 | 0.1 ± 0.7 | 0.3 ± 2 | 0.1 ± 0.9 | 0.4 ± 2.5 | 0.2 ± 1.2 | 0.4 ± 2.5 | 0.1 ± 1.1 | 0.3 ± 2.1 | 0.1 ± 1.1 | 0.4 ± 2.3 | 0.2 ± 1.1 | 0.4 ± 2.2 | 0.1 ± 1.1 |  |  |
| Offal | 1 ± 3.1 | 1.2 ± 3.1 | 0.8 ± 2.5 | 1 ± 2.5 | 0.6 ± 1.8 | 0.6 ± 1.6 | 0.7 ± 2.2 | 0.8 ± 2.1 | 0.9 ± 2.6 | 1 ± 2.5 | 0.9 ± 2.8 | 1.1 ± 2.7 | 0.8 ± 2.3 | 0.9 ± 2.3 | 0.8 ± 2.4 | 0.9 ± 2.4 | 0.8 ± 2.3 | 0.8 ± 2.2 |  |  |
| Delicatessen | 9.7 ± 6.6 | 8.1 ± 5.5 | 9 ± 5.8 | 7.5 ± 4.9 | 7.6 ± 4.9 | 6.3 ± 4.1 | 8.8 ± 5.7 | 7.3 ± 4.8 | 10.1 ± 6.7 | 8.5 ± 5.7 | 9.3 ± 6.1 | 7.7 ± 5.1 | 8.9 ± 5.8 | 7.5 ± 5 | 9.7 ± 6.3 | 8 ± 5.3 | 9.4 ± 6.1 | 7.8 ± 5.2 |  |  |
| ***Fish*** | 7.9 ± 8 | 10.2 ± 8 | 5.8 ± 6.1 | 7.5 ± 6 | 4 ± 4.6 | 5.1 ± 4.2 | 6.1 ± 6.6 | 7.7 ± 6.1 | 7 ± 7.4 | 9 ± 7.2 | 6.4 ± 6.6 | 8.2 ± 6.5 | 6.4 ± 6.8 | 8.2 ± 6.6 | 6.5 ± 6.9 | 8.3 ± 6.6 | 6.3 ± 6.6 | 8.2 ± 6.4 |  |  |
| ***Dairy products*** | 19.7 ± 9.9 | 22.4 ± 8.8 | 21.2 ± 9.8 | 23.9 ± 8.6 | 26.3 ± 11.1 | 29.6 ± 9.7 | 21.8 ± 10 | 24.4 ± 8.7 | 19.6 ± 9.6 | 22.2 ± 8.3 | 21.5 ± 9.9 | 23.9 ± 8.6 | 25.3 ± 11.5 | 28.3 ± 10 | 22.6 ± 10.5 | 25.5 ± 9.1 | 19 ± 9.1 | 21.4 ± 7.9 |  |  |
| Milk | 5.2 ± 5.9 | 6.6 ± 5.9 | 5.6 ± 6.1 | 7.1 ± 6 | 6.5 ± 7 | 8.3 ± 7 | 6.2 ± 6.7 | 7.7 ± 6.6 | 5.3 ± 5.9 | 6.6 ± 5.7 | 5.8 ± 6.2 | 7.2 ± 6 | 6.3 ± 7.1 | 8 ± 7.1 | 6.1 ± 6.7 | 7.6 ± 6.5 | 5.2 ± 5.7 | 6.6 ± 5.6 |  |  |
| Yogurts | 3.3 ± 4.3 | 5.1 ± 4.4 | 3.6 ± 4.5 | 5.4 ± 4.6 | 4.3 ± 5.4 | 6.7 ± 5.7 | 3.7 ± 4.6 | 5.6 ± 4.7 | 3.9 ± 4.8 | 5.7 ± 4.7 | 3.2 ± 4.1 | 4.8 ± 4.1 | 4.1 ± 5.1 | 6.2 ± 5.2 | 3.9 ± 4.9 | 5.9 ± 5 | 3.2 ± 4.1 | 4.9 ± 4.1 |  |  |
| Cheese | 10.8 ± 7.3 | 10.2 ± 6.4 | 11.6 ± 7.5 | 10.8 ± 6.4 | 15 ± 9.3 | 14 ± 8 | 11.5 ± 7.5 | 10.6 ± 6.3 | 10 ± 6.7 | 9.3 ± 5.7 | 12 ± 7.8 | 11.2 ± 6.7 | 14.5 ± 9.4 | 13.6 ± 8.1 | 12.2 ± 7.9 | 11.4 ± 6.9 | 10.2 ± 6.6 | 9.5 ± 5.7 |  |  |
| Other | 0.4 ± 0.3 | 0.5 ± 0.4 | 0.4 ± 0.4 | 0.5 ± 0.4 | 0.5 ± 0.5 | 0.6 ± 0.5 | 0.4 ± 0.4 | 0.5 ± 0.4 | 0.4 ± 0.4 | 0.5 ± 0.4 | 0.5 ± 0.6 | 0.7 ± 0.7 | 0.5 ± 0.5 | 0.6 ± 0.5 | 0.4 ± 0.4 | 0.6 ± 0.5 | 0.4 ± 0.3 | 0.5 ± 0.4 |  |  |
| ***Eggs*** | 3.7 ± 3.1 | 4.8 ± 3.4 | 3.7 ± 2.9 | 4.8 ± 3.1 | 2.8 ± 2 | 3.5 ± 2.1 | 4.7 ± 3.7 | 6 ± 4 | 3.4 ± 2.7 | 4.4 ± 3 | 3.8 ± 3 | 4.9 ± 3.2 | 3.5 ± 2.8 | 4.5 ± 3.1 | 3.8 ± 3.1 | 4.9 ± 3.3 | 3.3 ± 2.5 | 4.3 ± 2.7 |  |  |
| **Plant** | 19.8 ± 6.7 | 20.4 ± 6.1 | 30.8 ± 8.5 | 31.1 ± 7.8 | 37.9 ± 9.6 | 37.2 ± 8.6 | 29.9 ± 8.1 | 30.4 ± 7.4 | 24.2 ± 7.5 | 25.3 ± 7.1 | 29.4 ± 8.4 | 30.2 ± 7.5 | 25 ± 7.6 | 25.2 ± 7 | 26.2 ± 7.5 | 26.7 ± 6.9 | 30.8 ± 8.4 | 31.5 ± 7.7 |  |  |
| Cereals | 14.3 ± 5.7 | 13.7 ± 4.8 | 22.5 ± 7.7 | 21.2 ± 6.5 | 30.8 ± 9.5 | 28.4 ± 8.1 | 21 ± 7.2 | 19.8 ± 6.1 | 16 ± 6.1 | 15.2 ± 5.1 | 21.1 ± 7.4 | 19.8 ± 6.2 | 17.7 ± 6.6 | 16.5 ± 5.6 | 17.9 ± 6.4 | 16.8 ± 5.4 | 21.5 ± 7.4 | 20.2 ± 6.3 |  |  |
| Potatoes | 1.2 ± 0.9 | 1.4 ± 0.9 | 1.7 ± 1.2 | 1.9 ± 1.2 | 1 ± 0.7 | 1.1 ± 0.7 | 1.6 ± 1.1 | 1.7 ± 1.1 | 1.7 ± 1.2 | 1.9 ± 1.2 | 1.7 ± 1.2 | 1.9 ± 1.3 | 1.7 ± 1.2 | 1.8 ± 1.2 | 1.9 ± 1.3 | 2.1 ± 1.3 | 1.8 ± 1.2 | 1.9 ± 1.2 |  |  |
| Fruit | 0.6 ± 0.7 | 0.9 ± 1 | 1 ± 1.1 | 1.5 ± 1.6 | 1.7 ± 1.7 | 2.4 ± 2.1 | 1.4 ± 1.4 | 2 ± 1.9 | 1 ± 1.1 | 1.5 ± 1.6 | 1 ± 1.3 | 1.7 ± 2.1 | 0.6 ± 0.9 | 1 ± 1.4 | 1 ± 1.1 | 1.6 ± 1.5 | 1.6 ± 1.4 | 2.2 ± 1.9 |  |  |
| Vegetables | 1.7 ± 1.1 | 2.2 ± 1 | 2.3 ± 1.4 | 2.9 ± 1.4 | 1.8 ± 1.1 | 2.2 ± 1.1 | 2.5 ± 1.4 | 3.1 ± 1.5 | 2.6 ± 1.5 | 3.3 ± 1.6 | 2.5 ± 1.5 | 3.4 ± 1.7 | 2 ± 1.2 | 2.6 ± 1.3 | 2.4 ± 1.4 | 3 ± 1.5 | 2.4 ± 1.4 | 3.1 ± 1.5 |  |  |
| Nuts and seeds | 0.5 ± 0.8 | 0.5 ± 0.7 | 0.8 ± 1.5 | 0.8 ± 1.2 | 0.6 ± 1.1 | 0.7 ± 1.2 | 0.8 ± 1.4 | 0.8 ± 1.2 | 0.7 ± 1.2 | 0.7 ± 1 | 0.8 ± 1.3 | 0.8 ± 1.1 | 0.7 ± 1.3 | 0.7 ± 1 | 0.7 ± 1.2 | 0.7 ± 1 | 0.8 ± 1.5 | 0.8 ± 1.2 |  |  |
| Legumes | 0.5 ± 0.8 | 0.5 ± 0.9 | 1.2 ± 2.1 | 1.2 ± 2.1 | 0.8 ± 1.3 | 0.8 ± 1.4 | 1.3 ± 2.1 | 1.2 ± 2.1 | 1.2 ± 1.9 | 1.2 ± 2 | 0.9 ± 1.4 | 0.8 ± 1.5 | 1.1 ± 1.9 | 1.1 ± 1.9 | 1.1 ± 1.8 | 1.1 ± 1.8 | 1 ± 1.7 | 1.1 ± 1.9 |  |  |
| Other | 0.6 ± 0.8 | 0.8 ± 0.9 | 0.8 ± 1 | 1 ± 1.1 | 0.8 ± 1 | 1 ± 1 | 0.9 ± 1 | 1.1 ± 1.1 | 0.7 ± 0.9 | 1 ± 1 | 0.9 ± 1.1 | 1.2 ± 1.2 | 0.7 ± 0.9 | 0.9 ± 0.9 | 0.8 ± 1 | 1 ± 1.1 | 1.2 ± 1 | 1.5 ± 1.1 |  |  |
| Seasonings | 0.4 ± 0.3 | 0.5 ± 0.4 | 0.4 ± 0.4 | 0.6 ± 0.5 | 0.5 ± 0.4 | 0.6 ± 0.5 | 0.5 ± 0.4 | 0.6 ± 0.5 | 0.4 ± 0.4 | 0.6 ± 0.6 | 0.5 ± 0.4 | 0.6 ± 0.6 | 0.4 ± 0.4 | 0.5 ± 0.5 | 0.4 ± 0.4 | 0.5 ± 0.5 | 0.5 ± 0.4 | 0.6 ± 0.7 |  |  |

**Supplemental references**

1. Suga H, Murakami K, Sasaki S. Development of an amino acid composition database and estimation of amino acid intake in Japanese adults. Asia Pacific journal of clinical nutrition 2013;22(2):188-99.

2. Centre d’Information des Viandes & INRA. Internet: <http://www.lessentieldesviandes-pro.org/pdf/PDF-tous%20morceaux.pdf> (accessed 08/03/2017).

3. U.S. Department of Agriculture ARS. USDA Branded Food Products Database . Nutrient Data Laboratory Home Page, <http://ndb.nal.usda.gov>. 2016.

4. Subdivision on Resources, Council for Science and Technology, Ministry of Education, Culture, Sports, Science and Technology (MEXT). Standard Tables of Food Composition in Japan - 2015 - (Seventh Revised Edition). 2015.

5. Health Canada. Canadian Nutrient File - <http://www.hc-sc.gc.ca/fn-an/nutrition/fiche-nutri-data/index-eng.php>. 2015.

6. The New Zealand Institute for Plant & Food Research Limited. New Zealand Food Composition Database: New Zealand FOODfiles 2014 Version 01. Retrieved October 30, 2016 from <http://www.foodcomposition.co.nz/foodfiles>. 2015.

7. FAO/INFOODS. Guidelines for Checking Food Composition Data prior to the Publication of a User Table/Database-Version 1.0. 2012.

8. Joint FAO/WHO/UNU Expert Consultation on Protein and Amino Acid Requirements in Human Nutrition (2002 : Geneva S. Protein and amino acid requirements in human nutrition : report of a joint FAO/WHO/UNU expert consultation. World Health Organization, WHO Technical Report Series 2007;935:256.

9. Martin A, Touvier M, Volatier J-L. The Basis for Setting the Upper Range of Adequate Intake for Regulation of Macronutrient Intakes, Especially Amino Acids. The Journal of Nutrition 2004;134(6):1625S-9S.

10. Trumbo P, Schlicker S, Yates AA, Poos M. Dietary Reference Intakes for Energy, Carbohydrate, Fiber, Fat, Fatty Acids, Cholesterol, Protein and Amino Acids. Journal of the American Dietetic Association 2002;102(11):1621-30.
